# Cardiac rehabilitation may not provide a quality of life benefit in coronary artery disease patients

**DOI:** 10.1186/1472-6963-12-406

**Published:** 2012-11-19

**Authors:** Rosanna Tavella, John F Beltrame

**Affiliations:** 1 Cardiology Unit, The Queen Elizabeth Hospital, University of Adelaide, 28 Woodville Rd, Woodville South, South Australia, 5011, Australia; 2 Discipline of Medicine, University of Adelaide, Adelaide, South Australia, Australia

**Keywords:** Coronary artery disease, Cardiac rehabilitation, Health-related quality of life

## Abstract

**Background:**

Improvements in patient-reported health-related quality of life (HRQoL) are important goals of cardiac rehabilitation (CR). In patients undergoing coronary angiography for angina and with documented coronary artery disease (CAD), the present study compared HRQoL over 6 months in CR participants and non-participants. Clinical predictors of CR participants were also assessed.

**Methods:**

A total of 221 consecutive patients undergoing angiography for angina with documented CAD and who were eligible for a CR program were recruited. CR participants were enrolled in a six-week Phase II outpatient CR course (31%, n = 68) within 2 months following angiography and the non-participants were included as a control. At baseline (angiography), one and six months post angiography, clinical and HRQoL data were obtained including the Short Form-36 (SF-36) and the Seattle Angina Questionnaire (SAQ). The response rate for the HRQoL assessment was 68% (n = 150). Cross sectional comparisons were age-adjusted and performed using logistic or linear regression as appropriate. Longitudinal changes in HRQoL were assessed using least squares regression. Finally, a multiple logistic regression was fitted with CR participant as the final outcome.

**Results:**

At angiography, the CR non-participants were older, and age-adjusted analyses revealed poorer physical (angina limitation: 54 ± 25 versus 64 ± 22, p <0.05) and mental HRQoL (significant psycho-social distress: 62%, n = 95 versus 47%, n = 32, p <0.05) compared to the CR participants. In addition, the CR participants were more likely to have undergone angiography for myocardial infarction (OR = 2.8, 95% CI 1.5-5.3, p = 0.001). By six months, all patients showed an improvement in HRQoL indices, however the rate of improvement did not differ between the controls and CR participants.

**Conclusion:**

Following angiography, CAD patients reported improvements in both generic and disease-specific HRQoL, however CR participation did not influence this outcome. This may be explained by biases in CR enrollment, whereby acute patients, who may be less limited in HRQoL compared to stable, chronic patients, are targeted for CR participation. Further investigation is required so CR programs maximize the quality of life benefits to all potential CR patients.

## Background

Cardiac rehabilitation (CR) programs are recognized as an effective component for the management of patients with coronary artery disease (CAD) [1,2]. Two systematic reviews of almost 50 randomised controlled trials showed a 20% reduction in all cause mortality and a 27% reduction in cardiac mortality at two to five years [3,4]. However, the impact of CR on health-related quality of life (HRQoL) has been inconclusive [4] due to the limited studies and the methodologies employed.

Previous CR studies have largely targeted patients with a recent MI, coronary revascularisation or cardiac failure. However all patients with CAD could potentially benefit from CR, especially those with chronic diseases who are more likely to be significantly disabled. Despite this, it appears that patients with chronic angina are less likely to participate in these programs [5], especially if they are female [6] or do not undergo revascularisation procedures.

This study evaluated CAD patients undergoing diagnostic angiography to ascertain (i) the clinical characteristics and HRQoL of patients undergoing CR as compared to those who do not, (ii) to assess the clinical predictors of CR participants, and (iii) compare the 6-month HRQoL progress following angiography in those that do and do not receive CR.

## Methods

This prospective, observational study recruited patients undergoing diagnostic angiography at the Queen Elizabeth Hospital (QEH) (Adelaide, Australia) between April 2003 and May 2007 and followed their progress over a 6-month period. The QEH is a tertiary hospital that provides cardiac care to the North-Western suburbs of Adelaide as well as diagnostic angiographic facilities for rural communities and smaller metropolitan hospitals. The Hospital provides Phase II CR services only for those patients residing within its local health care jurisdiction. This study was approved by the Central Northern Adelaide Health Service Ethics of Human Research Committee.

### Study patients

Inclusion criteria for study enrollment included patients presenting for (i) invasive coronary angiography undertaken for the investigation of chest pain, (ii) angiographically documented obstructive CAD (≥ 50% stenosis in at least one epicardial coronary artery), and (iii) eligibility for Phase II CR at QEH (defined by residential location in the Western suburbs of Adelaide, Australia).

The study exclusion criteria were (a) non-coronary disorders such as aortic valve disease, and (b) inability to communicate in English/unable to complete written questionnaires.

### Cardiac rehabilitation program

The QEH Phase II CR program comprises of biweekly visits held over a 6-week period. The course incorporates counseling and education on overall heart health and risk factor identification, nutrition, smoking cessation and weight loss. In addition, relaxation techniques and stress management for both patients and their partners or carers are included. A structured exercise program with an individualized exercise prescription was incorporated into each session.

### Study protocol

Patients scheduled for coronary angiography for the investigation of chest pain were approached prior to the procedure and informed consent was obtained. At this time, a detailed clinical history was attained via standardized medical case-note review (conducted by clinical nurses and medical staff). At time of consent, all patients underwent a short interview with clinical nurses or trained research assistants assessing cardiovascular risk profile, and angina severity which was graded according to the Canadian Cardiovascular Society Classification (CCSC). This was followed by HRQoL assessment utilizing the self-administered Short-Form 36 (SF-36) [7] and the Seattle Angina Questionnaires (SAQ) [8] instruments. At 1 and 6 months post-angiography, patients were re-assessed by: (a) SF-36 and SAQ mailed out for completion, (b) review of hospital administrative system for hospital admissions/presentations and (2) follow-up phone calls made assessing angina status, medication use and clinical events.

### Parameters assessed

Participation in CR was strictly defined as completion of the Phase II CR program as identified by the hospital CR services medical record system. The CR records were reviewed to identify patients who were documented as having completed the CR program at the hospital. The CR records at a second hospital, outside the local health care jurisdiction, were also reviewed to verify if any patients included in the study underwent CR at another nearby facility. Clinical data was obtained by medical case note review and patient interview. Socio-economic status was defined by the Australian Bureau of Statistics’ Socio-Economic Indexes for Areas scores, an accepted proxy measure for socio-economic status based on regional analyses in Australia [9]. HRQoL was assessed using both a generic (SF-36) and disease specific (SAQ) questionnaire. The SF-36 is a well-established measure [7], and has been shown to have good reliability, validity and responsiveness in patients with CAD [10], and also in a CR setting [11]. Elevated psychosocial distress was identified by the SF-36 Mental Summary Score (MCS) of ≤45 [12]. The SAQ is a disease-specific functional status measure quantifying five clinically relevant domains related to angina: physical limitation, angina stability, angina frequency, treatment satisfaction & quality of life [8]. It is reported to be the most appropriate disease-specific instrument for CAD [13].

### Statistical analysis

Comparisons were made between patients who completed the Phase II CR program and those who did not participate in the program. Cross-sectional analyses between the CR participants and non-participants, for categorical or continuous variables were performed using either logistic or linear regression respectively, and age adjusted where appropriate. Longitudinal changes in SF-36 and SAQ scores from baseline to 6 months between the CR groups were compared utilising either random effects generalised least square or fixed effects ordinary least squares regression [14]. A multiple logistic regression was fitted with CR attendance as the final outcome. Variables identified for the multivariate analysis were selected following univariate tests significant at a *p* value level of 0.25. The final model was obtained using automatic backward elimination, and the final odds ratios (ORs) with 95% CIs and *p* values are reported. All analyses were performed with STATA (Version 11, StataCorp, Texas, USA).

### Missing HRQoL data management

Follow-up HRQoL data over the 6 months was available in 68% of patients undergoing coronary angiography. To account for possible missing value bias, multiple imputation techniques [15] were used, conditioned upon the available SAQ and SF-36 scores. Imputation was performed by chained equations utilising switching regression, an iterative multivariable regression technique [16]. The data analysis was then repeated using the above-described methods but no significant deviation from the original models were observed. The HRQoL results presented below are those from the imputed data analysis.

## Results

**Figure 1:**
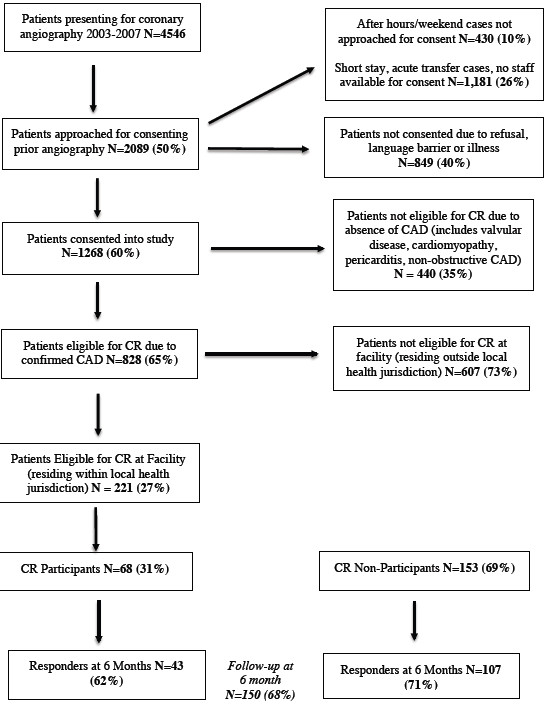
**Flow Chart Describing Patients Presenting for Coronary Angiography and Eligible for Cardiac Rehabilitation at the Queen Elizabeth Hospital.** Study flow diagram describing (**a**) the number of patients undergoing coronary angiography during the recruitment period, (**b**) the number of patients who were approached for consent, (**c**) reasons for no consent and exclusion, (**d**) the number of patients with confirmed CAD, (**e**) the number of patients who were eligible for CR program and (**f**) the response rate of CR participants and non-participants.

Between 28th April 2003 and 11th April 2007, 828 patients with angiographic evidence of CAD were identified and consented. Of these, 221 (27%) were eligible for referral to TQEH Phase II cardiac rehabilitation services and were recruited into the study. Figure 1 shows the Study Flow Diagram. Of these patients, 68 (31%) participated and completed the Phase II CR with almost 70% (n = 46) commencing the program within one month of coronary angiography. The mean waiting time for enrolment was 1.7 months (SD = 2.15, 95% CI 1.16-2.22).

### Baseline characteristics of study patients

#### Demographic and clinical features

Patients attending Phase II CR were significantly younger compared to patients who did not attend CR and thus subsequent analyses were age-adjusted. In relation to socioeconomic status, 24% (n = 37) of CR patients were identified as belonging to low, which was comparable to 16% (n = 11) in the CR non-participants (Table 1). The CR patients were more likely to have undergone angiography following an admission with an acute coronary syndrome (ACS) (Table 2), with 41% (n = 62) having experienced a recent myocardial infarction compared with 17% (n = 12) who did not have CR (p < 0.001). Despite this, the extent of angiographic disease was similar between groups as was the initial therapy although there was a slight trend towards medical therapy for those not participating in CR (Table 2).

**Table 1:** Baseline clinical characteristics between CR participants and non-Participants

	***n***	**No CR n = 153**	***n***	**CR n = 68**		**Total N = 221**
		**%/Mean ± SD**		**%/Mean ± SD**		**%/Mean ± SD**
**Cardiovascular Risk Factors**						
Age (years)	153	65 ± 12*	68	60 ± 12**	221	63 ± 12
Female	153	31%	68	24%	221	29%
Current Smoker	148	18%	68	21%	216	19%
Ex-smoker	147	45%	68	34%	215	41%
Hypertension	152	63%	67	55%	219	61%
Diabetes Mellitus	149	33%	67	28%	216	31%
Hypercholesterolaemia	149	74%	68	71%	217	73%
**Co-morbidities**						
Previous Myocardial Infarct	152	32%	68	32%	220	32%
Prior Revascularisation	153	28%	68	24%	221	27%
Cerebrovascular disease	149	10%	63	2%	212	8%
Chronic obstructive pulmonary disease	149	28%	63	13%*	212	24%
Peripheral arterial disease	149	7%	63	3%	212	6%
Gastroesophageal disorders	149	41%	65	32%	214	38%
Musculoskeletal disease	149	14%	63	22%	212	17%
Psychiatric disorders	147	25%	64	17%	211	23%
**Indication for Angiography**						
Recent Acute Coronary Syndrome	150	41%	66	65%**	216	49%
Chronic Stable Angina	150	55%	66	33%**	216	51%
**Low Socioeconomic status**	153	24%	68	16%	221	22%
**CCS II-IV at Angiography**	141	61%	63	51%	204	58%

** p < 0.01.

*p < 0.05, age adjusted.

**Table 2:** Baseline cardiac history & HRQoL characteristics between CR participants and non-Participants

	**n**	**No CR (n = 153)**	**n**	**CR (n = 68)**
**Pre-Angiography Cardioprotective Therapy**				
Anti-platelet	153	84%	68	82%
Beta-blockers	153	35%	68	19%
Statins	153	67%	68	66%
ACE-inhibitor	153	42%	68	40%
Angiotensin Receptor Blocker	153	18%	68	13%
**Obstructive CAD Findings**				
Single Vessel Disease	153	29%	68	32%
Double Vessel Disease		39%		32%
Triple Vessel Disease		32%		35%
**Initial Treatment Strategy**				
Medical Therapy	153	56%	68	41%
Coronary Angioplasty/stent		30%		43%
Coronary bypass graft		14%		16%
**Seattle Angina Questionnaire**				
Physical Limitation	153	54±25	68	64±22*
Angina Stability	153	37±31	68	40±34
Angina Frequency	153	59±27	68	66±27
Treatment Satisfaction	153	90±16	68	89±18
Quality of Life	153	42±22	68	44±24
**Short-Form 36**				
Physical Summary Score	153	34±11	68	38±11
Mental Summary Score	153	42±11	68	43±11
Elevated Psychosocial Distress	153	62%	68	47%*

*p < 0.05, **p < 0.01, age adjusted.

#### HRQoL indices

Scores on the HRQoL indices indicated the CR patients had a better quality of life in terms of both general health status and angina-specific morbidity at baseline (Table 2). Scores on the physical limitation domain of the SAQ were significantly higher in the CR patients compared to CR non-participants. The angina frequency domain was also higher (indicating less angina) in the CR patients, however this was not statistically significant. Consistent with this, scores on the SF-36 PCS tended to be higher in the CR patients. In addition, elevated psychosocial distress as identified by the SF-36 MCS, was lower in the CR patients (Table 2).

#### Predictors of CR participation

Predictors of CR participation were assessed in the overall group of patients presenting for angiography who were potentially eligible for referral. Following univariate analysis, backward elimination retained the following predictors: age, recent myocardial infarction and a prior history of musculoskeletal disease. In the final model, only recent myocardial infarction (OR = 2.8, 95% CI 1.5-5.3, p = 0.001) remained as a significant predictor of CR participation.

### Six month outcomes in study patients

#### Clinical outcomes

At 6 months following angiography, at which time the CR patients had completed their rehabilitation programme, there were no differences between groups in relation to cardiac events (Table 3). Furthermore there were no differences between groups in changes in medical therapy or angina limitations as assessed via the CSCC system (Table 3).

**Table 3:** Clinical progress over 6 months following angiography

	***n***	**No CR**	***n***	**CR**	***n***	**Total**	***p***
		**%**		**%**		**%**	**Age adjusted**
**CCS Class II-IV 6 Months**	78	17%	34	21%	112	18%	0.593
**Medication Changes**							
Any Changes	126	68%	60	68%	186	68%	0.965
Started Nitrates	125	22%	60	25%	185	23%	0.562
Started CCB	126	22%	60	13%	186	19%	0.165
Started Beta Blocker	126	14%	60	13%	186	14%	0.929
Started Any Anti-anginal	126	44%	60	45%	186	44%	0.757
**Cardiac Endpoints**							
ED Chest Pain Presentation	128	16%	59	10%	187	14%	0.263
Chest pain re-admission	127	16%	59	10%	186	14%	0.345
Myocardial infarction	127	2%	59	0%	186	2%	
Repeat angiography	127	4%	59	3%	186	4%	0.646
PCI	126	13%	60	20%	186	15%	0.401
CABG	126	24%	59	22%	185	23%	0.905
Mortality	153	2%	68	1%	221	2%	0.844
MI/Mortality	153	3%	68	1%	221	3%	0.659

#### HRQoL indices

**Figure 2:**
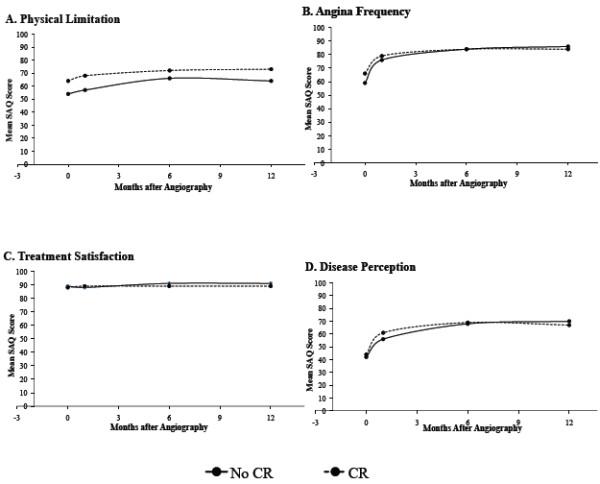
**A-D. SAQ Domain Scores Over Six Months in CR Participants and Non-Participants.** SAQ domain scores from baseline to six months compared between cardiac rehabilitation participants and non-participants. Longitudinal analysis revealed no significant differences in the rate or extent of improvement in the scores between CR participants and non-participants.

**Figure 3:**
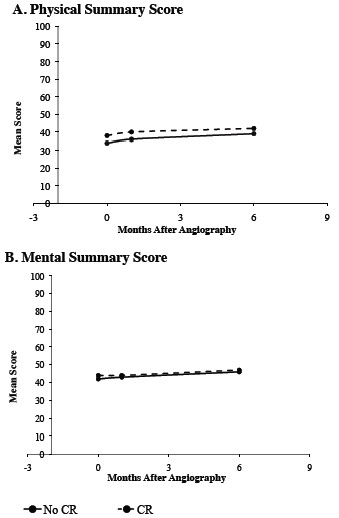
**A and B.SF-36 Summary Scores Over Six Months.** SF-36 Physical (**A**) and Mental Summary Score (**B**) from baseline to six months compared between cardiac rehabilitation participants and non-participants. Longitudinal analysis revealed no significant differences in the rate or extent of improvement in the scores between CR participants and non-participants.

Figures 2 and 3 summarise the HRQoL indices over the 6-month period for the 2 study groups. The HRQoL indices were measured at 1 and 6 months although at the 1-month assessment time point, only 70% (n = 46) of the CR participants had commenced the program and none had completed the 6-week program. By 6 months, all the CR participants had completed the CR program. The SAQ-derived physical limitation, angina frequency, and quality of life scales all showed significant improvement over the 6-month study period. Both study groups also demonstrated statistically significant improvements in the SF-36 PCS and MCS over the 6-month period. However, no differences were observed in the rate of improvement from baseline over 6 months between the two groups in SAQ and SF-36 scores (Figures 2 and 3). Furthermore, cross-sectional analysis on the 1 and 6 month endpoints revealed no significant differences for both the SAQ and SF-36 scales between the two groups.

## Discussion

This case–control study assessed the clinical characteristics of patients with angiographically-documented CAD who underwent CR and assessed their subsequent clinical and HRQoL progress over a 6-month period. The key findings from this study are (i) patients who undertake CR are younger and most likely to have experienced a recent myocardial infarction; and (ii) in the 6-month follow-up period, all patients showed an improvement in HRQoL indices independent of whether or not they had CR with no significant incremental benefit found in those who did have CR.

### Patient selection for cardiac rehabilitation

Typically, CR has been targeted towards CAD patients who have experienced a recent MI [17] or undergone revascularisation therapies [18]. In this study, two-thirds of the CR patients had experienced a recent ACS, and this was the defining clinical characteristic of the cohort. Although it is paramount that all patients with an ACS be offered CR, it is equally important that stable patients with documented CAD should be offered CR. Indeed, as shown in our study, those who did not undertake CR were more chronically disabled, older, more physically disabled, more frequently identified with psychosocial distress and more often had a history of chronic stable angina (Table 1). Hence those who did not undertake CR may potentially have benefited more than those who did. Unfortunately this study does not provide insights into why patients did/did not participate in the Phase II CR program, thus it may reflect a selection bias by the health care system or patient choice.

Regardless of the reason, the findings of this study suggest that patients with chronic stable angina need to be targeted for enrolment to a CR program and this may be best undertaken at the time of diagnostic angiography. Targeting CR to patients undergoing revascularisation therapies is important and will provide CR to some chronic stable angina patients. However this study showed that those who did not receive CR tended to be managed with medical therapy (Table 2). Moreover, CR in stable angina patients has been shown to be superior to percutaneous coronary intervention in increasing exercise capacity and reducing hospital readmissions [19]. Thus an active program to target CR for disabled chronic stable angina patients needs to be considered.

### The impact of cardiac rehabilitation on HRQoL

Improvement in HRQoL following CR has not been consistently documented as highlighted in a meta-analysis, which demonstrated that improvement in HRQoL with CR was better than in controls in only two of 12 trials [4]. However, comparing HRQoL findings in CR research is difficult due to the diversity of the instruments used. Earlier studies often used psychosocial well-being measures [5,6,20] rather than established generic and disease-specific instruments. Although more recent trials have increasingly employed the SF-36, there are still conflicting accounts concerning the influence of CR on HRQoL [21-24]. In addition, only few studies have used a disease-specific measure, however the choice of instrument has varied, and have included the MacNew [25,26], the Cardiac Quality of Life Index [27], and Quality of Life after Myocardial Infarction [28]. Hofer et al [29] highlights that a consistent application of a single disease-specific measure is warranted in CR research. Although the SAQ is an established and arguably the best disease-specific measure for CAD [13], the present study is the first to utilize the SAQ in a CR setting.

### Study implications for cardiac rehabilitation

The failure of CR to impact on HRQoL in this and other studies warrants further consideration as it may reflect nuances relating to these studies rather than CR being ineffective. These include factors relating to the patient, the CR therapy and the study design.

#### Patient factors

As discussed above and reported in previous studies [30], the patients who did not participate in CR were the most disabled and thus may well have been those that would have derived the most benefit. Five patient-related factors may have influenced the findings in this study and should be closely considered in further studies. Firstly, CR efficacy in relation to impact on HRQoL has been shown to be age-dependent with previous studies reporting that younger (i.e. <41 years) and older (>65 years) patients benefitting the most from CR with no benefits observed in those 41–65 years [21,29]. In this study, the average age of the CR participants was 60 years, which may have influenced the findings. Secondly, almost half of the CR patients were identified as experiencing elevated psychosocial distress and this has a major impact on HRQoL. Thus if the CR program also focused on the treatment of psychosocial distress, an incremental improvement in HRQoL may have also been achieved. Thirdly, the CR study population predominantly underwent this therapy following a recent myocardial infarct. Muller-Nordhorn et al [21] showed that only patients undergoing CR following revascularisation procedures showed improvement in their SF-36 scores whereas recent myocardial infarct patients did not. Fourthly, patient participation in CR was 31%, which is comparable to rates reported by other studies, which have shown 14-43% of potential cardiac patients participate in CR programs [31-33]. Although all the study patients were eligible for CR, why the non-participants did not avail themselves to this opportunity is unclear. The focus by the health workers on the acute presentation [34,35] may have under-emphasized the importance in chronic conditions and thus education of both health care workers and patients is required. Logistic factors may have also influenced patient participation, such as transport availability/cost and session times. Finally, although all the CR patients completed the program, the commitment they made to the therapy was not assessed.

#### The CR therapy

CR programs are heterogeneous in content although professional societies are developing guidelines with core components to standardize the therapy [36,37]. Hence the findings from this study may not be applicable to other CR programs. In particular, tailored programs for different CAD presentations or populations may be useful to maximize benefits for patients, as suggested by Piepoli et al [38]. For example, tailored gender-specific programs have been shown to provide increased quality of life improvement for women [39]. Other aspects that require further considerations include CR program delivery and duration. The hospital-based CR program in this study may be better delivered as a telephone or on-line program, which may assist in patient participation and provide long-term access to all. This is important since recent studies have shown that a continued intervention following CR is effective in improving cardiovascular outcomes [40,41]. Also the duration of the CR program in this study was only 6 weeks whereas Piepoli [38] highlights that such short-term approaches are unlikely to yield long-term benefits or impact on quality of life.

#### Study design

The current study was a longitudinal case–control design and the limitations of this design may have influenced the findings. Firstly, the non-randomised allocation to CR therapy may have been influenced by a selection bias although the two groups were similar in their baseline HRQoL indices. Secondly, the study period was limited to 6 months and longer follow-up periods may have influenced the results. Of note, a meta-analysis of CR therapies showed improvement in all-cause mortality only at 3-year follow-up and not earlier [42]. Thirdly, important information such as referral and drop out rates, reasons for non-inclusion in CR, such as ambulation, were not available in this study. The lack of standardized data collection and thus insufficient program information causes difficulty when assessing biases [43]. Lastly, although the study was powered to detect a difference of 6 points on the SF-36 PCS, the total sample size was relatively small; larger studies are required and should investigate smaller but clinically relevant differences in HRQoL. It should also be noted that HRQoL assessment is best undertaken by patient interview where possible as opposed to mail-out surveys to increase response rate.

## Conclusion

The importance of CR in reducing cardiac events amongst patients with established CAD has been previously established, however its benefit in improving HRQoL is less clear and has not been affirmed in this study. This study has shown that those CAD patients who do not undergo CR are usually more disabled with chronic disorders and thus may potentially be those who would derive the most benefit from this therapy. Thus further studies are required to identify which patients will receive the optimal benefit from CR therapy rather than simply targeting those who have experienced a recent ACS.

## Competing interests

The authors declare that they have not competing interests.

## Authors’ contributions

JB conceived the study, and participated in its design and helped draft the manuscript. RT participated in the study design, carried out the patient assessments, performed the statistical analysis and drafted the manuscript. Each author has read and approved the final manuscript.

## Pre-publication history

The pre-publication history for this paper can be accessed here:

http://www.biomedcentral.com/1472-6963/12/406/prepub
